# Effects of the COVID-19 pandemic on working conditions and mental health among employees in health and social services

**DOI:** 10.3389/fpubh.2026.1899434

**Published:** 2026-08-31

**Authors:** Claudia Peters, Madeleine Dulon, Jan Felix Kersten, Christofer Hartung, Albert Nienhaus, Anja Schablon

**Affiliations:** 1 Institute for Health Services Research in Dermatology and Nursing, University Medical Center Hamburg-Eppendorf, Hamburg, Germany; 2 Department of Occupational Medicine, Hazardous Substances and Public Health, German Social Accident Insurance Institution for the Health and Welfare Services, Hamburg, Germany

**Keywords:** COVID-19, health personnel, mental health, post-acute COVID-19, work ability

## Abstract

**Background:**

The COVID-19 pandemic has posed major challenges in the workplace. This exploratory, cross-sectional study investigated the association between occupational activities and mental health.

**Methods:**

In 2023, 576 employees from health and social care institutions were surveyed about their occupational situation, their self-assessed ability to work, health-related issues, as well as their mental health and quality of life.

**Results:**

The respondents were predominantly women with an average age of 49 years. Of these, 59% were healthcare workers (HCWs), such as nurses, physicians and therapists. The non-healthcare group mainly consisted of people working in educational or care-related professions, as well as administrative staff. In comparison, HCWs reported poorer work ability (mean 6.9 vs. 7.3) and described a work situation characterized by emotional strain, time pressure and work-privacy conflict. They were also more likely to consider changing jobs or leaving their profession. Around one-fifth of all respondents suffered from moderate to severe mental distress. HCWs also reported symptoms of depression more frequently and a lower quality of mental life.

**Conclusion:**

Employees in medical professions were under considerable strain due to their work during the pandemic. Therefore, the prevention of high levels of mental stress should play a key role in maintaining and strengthening long-term mental health.

## 1 Introduction

The COVID-19 pandemic had considerable impacts on all areas of life and brought about profound changes. This period also posed exceptional challenges for employees in the workplace. At the beginning of the pandemic, employees in personal service occupations and healthcare professions in particular had a higher risk of infection than other occupational groups (1, 2) as well as an increased risk of hospitalization (3, 4). By the end of 2023, 413,077 suspected cases of occupationally acquired COVID-19 infection had been reported to the German Social Accident Insurance Institution for the Health and Welfare Services; more than 200,000 reports were recorded in 2022 alone. Employees in residential geriatric care and childcare as well as staff from hospitals and medical practices in particular were affected (5).

The effects of the pandemic on mental health were already observed at an early stage. During this period, mental health among the general population decreased considerably while symptoms of depression and anxiety increased (6). The increase also continued into the later phase of the pandemic (7). As early as 2020, it became apparent that healthcare personnel frequently reported psychosocial problems (8). Among healthcare workers (HCWs), higher prevalence of burnout, anxiety, depression and stress disorders were observed than in the general population (9). High psychological strain, which further increased over the course of the pandemic, was reported for personnel involved in direct patient care (10). Other studies described the experience of stress caused by traumatic events and by working with COVID-19 patients (11); this manifested itself primarily through symptoms of depression and anxiety (12, 13). Reviews also confirmed that the pandemic led primarily to anxiety, depression and sleep problems among numerous employees in the healthcare sector (14, 15). Fear of infecting loved ones as well as occupational insecurity, stigmatization, and uncertainty regarding the extent, duration and effects of the pandemic were major concerns faced by healthcare personnel (16). In addition, concerns about contracting SARS-CoV-2 at work as well as feeling overwhelmed by an increased workload were highlighted as particular pandemic-related stressors for employees in the health and social care sector (17).

The aim of the present study was to describe the working conditions of employees in health and social care institutions 3 years into the COVID-19 pandemic and to compare the situation of medical staff with that of other occupational groups.

To this end, the following questions were to be answered:

a. Are the working conditions for HCWs different from non-medical professions?
b. How has the changed situation compared to the pre-pandemic period affected work ability?
c. What are the working conditions like for employees?
d. What is the state of mental health, including depression and anxiety, as well as health-related quality of life?

## 2 Methods

### 2.1 Participants

As part of an exploratory, cross-sectional survey, 3,000 randomly selected individuals insured by the German Social Accident Insurance Institution for the Health and Welfare Services (BGW) were surveyed. These individuals had reported workplace accidents in 2020 and 2021 that resulted in no prolonged incapacity for work, the sample can be considered a random sample drawn from the total population of employees insured by the BGW. The BGW insures employees from non-governmental institutions in the health and welfare sectors in Germany. In addition to healthcare professions, insured individuals also include social workers and other employees in health or social care institutions. For the survey, insured individuals from two regional administrations were selected and invited to participate in the study in April 2023. Following detailed information about the study, the insured individuals provided written consent for voluntary participation. The Ethics Committee of the Hamburg Medical Association approved the conduct of the study (2021-10463-BO-ff). For the analysis, insured individuals under 18 years of age and those with pension status were excluded (*n* = 17).

### 2.2 Variables and measures

An extensive questionnaire in paper-and-pencil format was developed for the survey (see Supplementary Table 1). In addition to sociodemographic information, the main section included questions on occupation, work area and occupational situation. For this purpose, work ability at the time of the study and before the pandemic was subjectively assessed on a scale of 0 (very poor) to 10 (very good), and its relation to physical and psychological job demands was evaluated (Work Ability Index - WAI) (18). The German version of the Copenhagen Psychosocial Questionnaire (COPSOQ) (19) was used to assess the relationship between work and private life, as well as emotional demands. Social support from colleagues and supervisors was examined using the SALSA questionnaire (20). Work organization in relation to time pressure was assessed for the periods before and during the pandemic (19). Another focus was on health-related aspects, including questions on personal infection history, frequency of SARS-CoV-2 infections, course of disease, persistent post-infection symptoms, and the number of COVID-19 vaccinations. Physician-diagnosed pre-existing conditions were also surveyed (18). To evaluate mental health, data on symptoms of anxiety and depression were collected using the Patient Health Questionnaire-4 (PHQ-4). The short form of the questionnaire covering two core criteria each for depressive disorders and generalized anxiety disorder (21). The value ranges of both subscales comprise scores between 0 and 6, whereby a result of ≥3 indicates symptoms of depression or an anxiety disorder. A total score of 0–12 points can be achieved. Scores of 0–5 indicate no or low psychological distress, 6–8 moderate distress, and 9–12 severe psychological distress. The PHQ-4 total scale showed good internal consistency (Cronbach's α = 0.86; 95% CI 0.84–0.88). The Veterans RAND 12-Item Health Survey (VR-12) (22) was used to assess health-related quality of life in relation to physical and mental health. This scale comprises 12 items combined into a physical and a mental summary scale, each ranging from 0 to 100. Higher scores are indicative of a better health related quality of life (HRQoL). The questionnaire was adapted for the German population and is considered to be an equivalent instrument to the SF-12. Internal consistency was good for the item sets underlying the physical (α = 0.87; 95% CI 0.85–0.89) and mental (α = 0.87; 95% CI 0.85–0.89) component scores of the VR-12.

### 2.3 Statistical analysis

Descriptive findings are presented as absolute and relative frequencies for categorical variables and as means and measures of dispersion for continuous variables. In each case, the proportions of categorical variables refer to the valid observations available. For the analysis, respondents were divided into two groups according to their occupational activity. The healthcare group, which consisted of medical, therapeutic and care professions, was compared with the group of non-medical professions. For comparison of groups of categorical data Fisher's exact test was performed, for ordinal variables with more than two categories, the Cochran–Armitage test was used. Student's *t*-test was applied for continuous outcome variables. To identify variables associated with binary outcomes, logistic regression analyses were conducted using a backward elimination procedure to obtain a parsimonious model: non-significant variables were removed from the model, starting with those with the highest *p*-value, so that, ultimately, apart from age and sex, only statistically significant predictors remained in the model. For epidemiological reasons, the main effects of age and sex were always included in the model regardless of their significance. The interaction of these two latter factors was taken into account in the backward selection procedure. Likelihood-ratio tests were used for backward elimination, and model fit is summarized using Nagelkerke's *R*^2^. Multicollinearity among the associated variables was assessed using variance inflation factors (VIFs), with values under 5 considered indicative for no problematic multicollinearity. The statistical significance level was set at α = 0.05 without correction for multiple testing. The analyses were conducted using SPSS version 29 and the programming language R (version 4.5.1).

## 3 Results

A total of 576 insured individuals participated in the survey (response rate 19.2%). The participants were predominantly women with a mean age of 49 years. The HCW group consisted of care staff (51.5%), physicians (16.2%), therapists (13.5%) and medical assistants (18.8%), working primarily in hospitals (43.5%), medical practices (29.1%) and residential geriatric care facilities (10.6%). The non-medical group consisted of employees in educational or caregiving professions (44.9%), administrative staff (36.9%) and domestic services staff (10.2%), working primarily in educational or social institutions (36.9%) as well as in hospitals (21.3%) and medical practices (9.8%) (no table). Around 50% of the employees in both groups were employed full-time; 65.3% of the medical personnel and 54.7% of the non-medical personnel had been practicing their profession for at least 10 years (Table 1). Approximately 50% of the respondents lived in a household with adults, and around 33% lived in a household with children. Between 16.8% of HCWs and 21.4% of the comparison group were single. Around 17% of the respondents were current smokers, 21.5% were obese, and 70% reported at least one physician-diagnosed pre-existing condition. No statistically significant differences were observed regarding COVID-19 infection history: more than 80% had experienced a SARS-CoV-2 infection at some point and more than 90% had been vaccinated. However, 45.4% of medical personnel reported at least one persistent symptom following COVID-19 infection, compared with 34.9% of the comparison group.

**Table 1:** Characteristics of the study population, separated by medical and non-medical professions.

Variable	HCWs		non-HCWs		*p*-value
	n = 340	59.0%	n = 236	41.0%	
Sex					
Female	305	90.0	207	88.1	0.496
Male	34	10.0	28	11.9	
N.A.	1		1		
Age in years (mean ± SD)	47.5 ± 12.7		51.2 ± 11.2		< 0.001
N.A.	0	1	
Working full-time	170	50.0	117	50.2	1.000
Employed since					0.023
0–5 years	63	18.5	54	22.9	
5–10 years	52	15.3	46	19.5	
>10 years	222	65.3	129	54.7	
N.A.	3		7		
Housing situation					
Adults without children	163	48.1	112	47.8	0.314
Adults with children	119	35.1	72	30.8	
Single	57	16.8	50	21.4	
N.A.	1		2		
Smoker	60	17.8	36	15.4	0.311
Obesity (BMI ≥ 30 kg/m^2^)	72	21.6	49	21.3	1.000
Pre-existing diseases	238	70.0	165	69.9	1.000
COVID-19 infection	291	85.6	195	82.6	0.645
Persistent symptoms following COVID-19	132	45.4	68	34.9	0.024
COVID-19 vaccination	314	92.4	221	94.8	1.000

N.A, not applicable.

### 3.1 Work ability

Subjectively assessed current work ability showed lower mean values among medical professionals than among other professions (6.9 vs. 7.3). However, both groups reported substantially better work ability before the COVID-19 pandemic (HCWs + 1.7 vs. non-HCWs + 1.2). Employees in medical professions assessed their work ability in relation to both physical (*p* = 0.021) and psychological demands (*p* = 0.098) less favorably than employees in non-medical professions (Table 2).

**Table 2:** Work ability comparing medical and non-medical professions.

Work ability	Value	HCWs (Mean ±SD)	non-HCWs (Mean ±SD)	*p*-value
General work ability				
Currently	0–10 (unfit for work - best work ability)	6.9 ± 2.3	7.3 ± 2.1	0.020
Before the pandemic		8.6 ± 1.6	8.5 ± 1.9	0.645
Work ability in relation to job demands				
Physical demands	1–5 (very good–very poor)	2.43 ± 0.96	2.24 ± 0.95	0.021
Psychological demands	1–5 (very good–very poor)	2.68 ± 1.01	2.53 ± 1.05	0.098

Compared with the pre-pandemic situation, 28.7% of the respondents reported a reduction in work ability of at least three points on the 0–10 scale (no table). Both age and group affiliation were significantly associated with reporting this reduction (Table 3). Age and group affiliation were independently associated with the outcome. VIF values were close to 1 for all variables (maximum VIF = 1.03), suggesting that there is no evidence of multicollinearity. The logistic regression model showed statistically significant associations; however, Nagelkerke's *R*^2^ was 0.043. When persistent post-infection symptoms were included in the model instead of COVID-19 infection itself, they were significantly associated with reduced work ability (OR 5.7; 95% CI 3.74–8.63).

**Table 3:** Logistic regression analysis of a reduction in work ability by at least three points.

Variable	Value	aOR	95% CI	*p*-value
Age	Per 10 years	1.18	1.00–1.40	0.048
Sex	Female	1.80	0.90–3.59	0.098
COVID-19 infection	–	1.70	0.94–3.09	0.081
Occupational group	HCWs	1.70	1.13–2.56	0.011

aOR, adjusted OR.

### 3.2 Work situation

**Figure 1:**
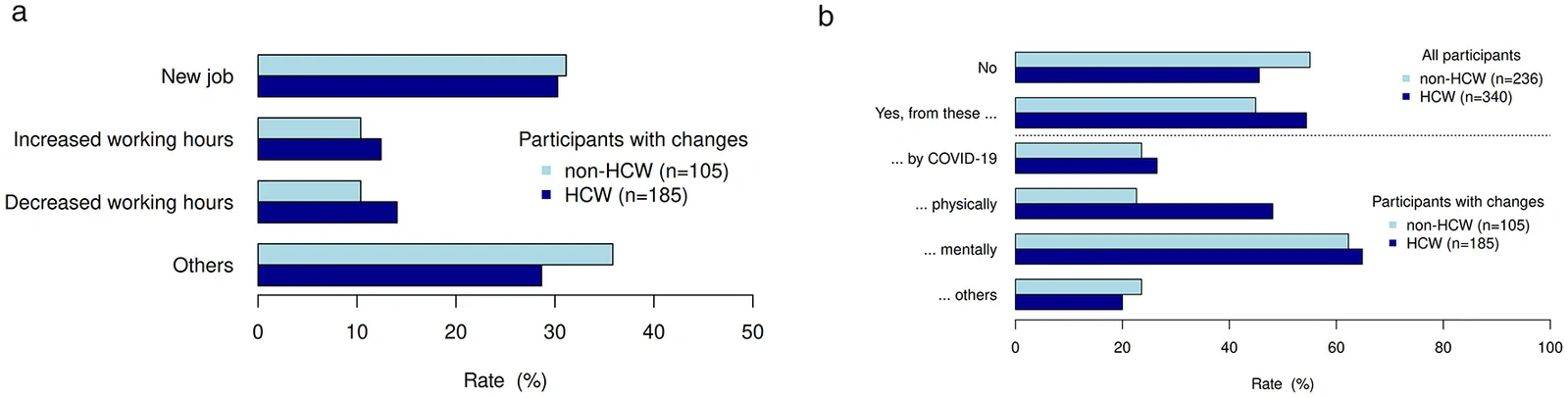
Changes (a) and the kind of changes (b) in the occupational situation since the beginning of the pandemic in 2020. (Multiple answers possible)

Since the start of the COVID-19 pandemic, 54.4% of the medical and 44.9% of the non-medical personnel reported a change in their occupational situation (*p* = 0.028). In this context, 64.9% cited high psychological work-related demands and 48.1% cited high physical work-related demands (*p* ≤ 0.001) as causes. In the comparison group, 62.3% reported psychological stressors and 22.6% physical demands as the most frequent causes of a change in their occupational situation (Figure 1a).

Approximately 33.3% of respondents reported having started a new role since 2020 as a change in their occupational situation. Approximately 25% reported a change involving either a reduction or an increase in working hours. Both groups primarily reported increased workload and greater stress, more difficult working conditions, an increased volume of work, staff shortages, and additional tasks and activities as further changes since the pandemic (Figure 1b).

Table 4 shows a comparison of different aspects to describe the work situation of both occupational groups. HCWs reported the relationship between work and private life as well as emotional demands to be statistically significantly worse for all individual aspects. Both groups assessed work-related time pressure as higher at the time of the survey than before the pandemic. Medical personnel reported poorer results (*p* < 0.001) regarding frequent work-related time pressure and decisions made under time pressure. Both groups reported insufficient time to complete tasks less frequently for the period before the pandemic than at the time of the survey. However, the differences between the occupational groups were not statistically significant for this aspect.

**Table 4:** Occupational aspects describing the work situation of medical compared with non-medical professions.

Item	Value	HCWs Mean ±SD	non-HCWs Mean ±SD	*p*-value
Work-privacy conflict			
Job demands interfere with private life	1–5 (disagree–fully agree)	3.16 ± 1.34	2.74 ± 1.27	< 0.001
Changing private plans because of work	1–5 (disagree–fully agree)	3.24 ± 1.38	2.67 ± 1.34	< 0.001
Work requires a great deal of energy, negative impact on private life	1–5 (disagree–fully agree)	3.37 ± 1.30	3.00 ± 1.35	0.001
Work takes up a great deal of time, negative impact on private life	1–5 (disagree–fully agree)	2.92 ± 1.31	2.59 ± 1.26	0.003
Emotional demands			
Work involves dealing with other people's problems	1–5 (never/hardly ever–always)	4.11 ± 0.97	3.89 ± 1.22	0.014
Work is emotionally demanding	1–5 (very low–very high)	3.96 ± 0.91	3.72 ± 1.11	0.005
Time pressure at work			
Frequent time pressure at work currently	1–5 (almost never–almost always)	4.11 ± 0.86	3.69 ± 1.04	< 0.001
Frequent time pressure at work before COVID-19 pandemic	1–5 (almost never–almost always)	3.83 ± 0.93	3.50 ± 1.02	< 0.001
Decisions under time pressure currently	1–5 (almost never–almost always)	3.68 ± 1.01	3.19 ± 1.13	< 0.001
Decisions under time pressure before COVID-19 pandemic	1–5 (almost never–almost always)	3.53 ± 0.99	3.04 ± 1.09	< 0.001
Too little time for all tasks –currently	1–5 (almost never–almost always)	3.51 ± 1.01	3.37 ± 1.16	0.153
Too little time for all tasks –before COVID-19 pandemic	1–5 (almost never–almost always)	3.21 ± 1.03	3.13 ± 1.12	0.352
Social support from colleagues and supervisors			
Colleagues are reliable	1–5 (almost never–almost always)	2.15 ± 1.22	2.06 ± 1.12	0.367
Discussion of problems with colleagues	1–5 (almost never–almost always)	2.43 ± 1.29	2.31 ± 1.30	0.280
Support from colleagues	1–5 (almost never–almost always)	2.60 ± 1.27	2.65 ± 1.30	0.624
Supervisors are reliable	1–5 (almost never–almost always)	2.46 ± 1.40	2.59 ± 1.51	0.289
Discussion of problems by supervisors	1–5 (almost never–almost always)	2.46 ± 1.40	2.47 ± 1.52	0.887
Support from supervisors	1–5 (almost never–almost always)	2.85 ± 1.40	2.96 ± 1.49	0.369
Change options in the last 12 months			
Thoughts of leaving profession	1–5 (never–daily)	2.37 ± 1.29	2.13 ± 1.28	0.029
Thoughts of changing jobs	1–5 (never–daily)	2.42 ± 1.34	2.19 ± 1.30	0.040

The results showed no substantial group differences regarding social support from colleagues and supervisors. Nevertheless, non-medical employees reported slightly greater reliability among colleagues as well as greater openness in discussing problems. By contrast, HCWs rated support from colleagues and supervisors, as well as the reliability of supervisors, more positively. The thought of leaving the profession (2.37 vs. 2.13) or changing jobs (2.42 vs. 2.19) was expressed considerably more frequently by medical than by non-medical personnel (Table 4).

### 3.3 Mental health

At the time of the survey, medical employees more frequently reported moderate to severe psychological distress (20.2 vs. 16.2%) (Table 5). Symptoms of depression were observed in 24.0% of HCWs compared with 17.5% among individuals in other professions. Symptoms of anxiety were reported by almost 25% of respondents in both groups.

**Table 5:** Mental health situation and health-related quality of life in medical compared with non-medical professions.

Variable	Value	HCWs	non-HCWs	*p*-value
Mental health				
		*n (%)*	*n (%)*	
No/low distress	0–5	268 (79.8)	191 (83.8)	0.257
Moderate distress	6–8	42 (12.5)	23 (10.1)	
Severe distress	9–12	26 (7.7)	14 (6.1)	
N.A.		4	8	
Symptoms of depression	≥ 3	81 (24.0)	40 (17.5)	0.075
Symptoms of anxiety	≥ 3	82 (24.3)	52 (22.7)	0.688
Health-related quality of life (HRQoL)				
		*Mean ±SD*	*Mean ±SD*	
Mental HRQoL	0–100	41.5 ± 12.3	43.5 ± 12.4	0.055
Physical HRQoL	0–100	43.3 ± 10.0	43.5 ± 10.3	0.812
N.A.		12	18	

N.A, not applicable.

The assessment of health-related quality of life with regard to mental aspects showed HRQoL among HCWs to be two points lower than among non-medical personnel (41.5 vs. 43.5). By contrast, there were no apparent differences between the groups regarding physical quality of life.

## 4 Discussion

This study examined, the effects of the COVID-19 pandemic on employees in health and social care institutions in a sample of 576 participants. Before the pandemic, the employees assessed their ability to work as better than it was at the time of the survey. HCWs reported poorer work ability than individuals in non-medical professions. Their work situation was additionally characterized by a high work–privacy conflict, strong emotional demands and high time pressure in everyday working life. They also considered changing jobs or leaving the profession more frequently. Almost 20% of all respondents reported moderate to severe mental distress. Symptoms of depression and a comparatively lower mental HRQoL were reported more frequently among medical personnel.

Other authors have also described increased occupational stress, reduced wellbeing, poorer mental health and impaired work ability among healthcare personnel as effects of the COVID-19 pandemic (23). In this context, work ability represents a widely discussed aspect in occupational studies, particularly in relation to COVID-19 infection and the resulting persistent symptoms. Numerous studies reported reduced work ability among individuals with Long COVID compared with individuals who had achieved complete remission or had not been infected (24–28). Our study also found that 84% of the respondents had been infected with SARS-CoV-2, and 35% continued to experience symptoms following infection. On average, participants reported lower work ability than before the pandemic. The multivariate analysis revealed a statistically significant association between Long COVID symptoms and a substantial reduction in current work ability. A study involving care staff observed a clear association between work ability and Long COVID. Low job satisfaction and low job control as well as high psychological demands and pre-existing conditions were identified as predictors (29). Kerksieck et al. (30) demonstrated the influence of physical and psychological occupational demands on work ability. Among individuals who had not recovered, greater health impairments were also associated with lower work ability in relation to physical and psychological demands. In our study, physical work demands in particular indicated poorer work ability among medical professions. In addition to medical treatment for SARS-CoV-2 infection, Braig et al. (31) described female sex, older age, and particularly physical work demands as risk factors for reduced work ability. In addition, there was evidence that a pre-existing mental health condition was associated with a greater reduction in work ability (26, 30). However, pre-existing conditions in general also showed considerable functional impairments such as low work ability and reduced work performance compared with HCWs without Long COVID and without pre-existing conditions (27).

Since the beginning of the pandemic, more than 50% of the medical personnel in our cohort reported a change in occupational situation. High physical (48%) and high psychological (65%) work-related demands were cited as reasons, which was also emphasized by the comparison group, consisting predominantly of employees in social and educational professions (62%). The change was associated either with a new role or altered working hours as well as increased workload, more difficult working conditions and/or staff shortages. Similar findings were reported in a systematic review of qualitative studies involving acute care staff. An increased workload due to the pandemic was reported; this was characterized particularly by staff shortages, longer shifts and less flexible working hours. Changed and additional tasks as well as insufficient support from supervisors and employers also contributed to the high level of strain (32). Pandemic-related work pressure among HCWs was also a major reason for deciding – or intending – to change jobs (33). In our study, the proportion of respondents intending to change jobs was higher in medical professions than in the comparison group. A meta-analysis showed that intentions to leave the profession among care staff and physicians are influenced primarily by personal factors such as age, professional experience, burnout symptoms and social support. Occupational determinants such as job demands, working conditions and corporate culture also influenced intentions to resign (34).

Working conditions can have different effects on the work situation. Analyses from the HealthyNurse Survey (35) showed that high occupational demands were associated with an increase in poor mental health characterized by feelings such as sadness, low mood and depression. Conversely, high job resources were associated with longer sleep duration and a reduced risk of excessive alcohol consumption. Our study examined work related demands by analyzing work–privacy conflict, emotional demands and working under time pressure. These items showed considerably poorer results for medical professions than for other professions. This was also reflected in reports of working under time pressure, which the HCWs surveyed had reported to a greater extent already for the period before the pandemic. A study among hospital staff identified emotional demands, increased workload, and time and performance pressure in particular as stress factors. By contrast, exchange with colleagues and mutual support were identified as important resources (36). Vivion et al. (37) also reported psychosocial workplace stressors, particularly high workload and high emotional demands, lack of recognition, low social support at work, and conflicts between work and private life. Quaglio et al. (38) described high workload and staff shortages as major problems among healthcare professionals in several EU countries. On the other hand, teamwork was described positively, particularly in terms of support in coping with occupational stress and maintaining work–life balance. A representative cohort study showed similar results: high support from colleagues, good leadership and control over work reduced stress among the respondents (39).

Almost 25% of the medical personnel reported symptoms of depression and anxiety. A similarly high proportion of symptoms of anxiety was found in the comparison group, whereas symptoms of depression were less frequent. Casjens et al. (40) reported an increase in mental distress during the pandemic among non-medical employees. Increased symptoms of depression and anxiety were also found among care staff (41, 42). By contrast, van der Noordt et al. (43) observed no relevant differences in mental health between HCWs and non-HCWs during the first year of the pandemic. Poorer mental health was apparent only during phases with high infection rates and strict measures. Health-related quality of life can be used to examine individual wellbeing and performance capacity on the basis of various criteria. A reduced quality of life among healthcare workers was already reported early in the pandemic, regardless of whether they had tested positive. (44). In our study, lower values among HCWs compared with non-medical professions were observed only for mental HRQoL. Numerous studies investigated HRQoL in connection with COVID-19. In particular, reduced quality of life was observed among individuals affected by Long COVID/Post-COVID (45–49).

### 4.1 Strengths and limitations

The present study addressed changes in occupational conditions resulting from the pandemic, despite COVID-19 infection and its consequences through Long/Post-COVID. Although these events cannot be considered entirely independent of the infection situation, we investigated primarily the effects of the pandemic on work ability and occupational situation.

We must assume that this study is subject to selection bias. The response rate of 19% is relatively low; though this is not unusual for surveys conducted in an occupational context. Nevertheless, it is possible that insured individuals whose professional lives and occupational situations had been particularly affected by the pandemic were more likely to participate. For example, this could be due to an increased workload and additional tasks against the background of existing staff shortages, which could ultimately be associated with an unsatisfactory situation. However, the low response rate may contribute to a non-responder bias if the participants differ systematically from those who declined to participate. The comparison group must also be considered a further limitation. This group includes a high proportion of employees in social and educational professions. It can be assumed that work areas involving direct contact with people and requiring an on-site presence rather than working from home share important characteristics and are consequently similar. A comparison between medical professions and other occupational sectors or the general population might therefore reveal more pronounced differences. As shown by various studies, the influence of COVID-19 infections and Long/Post-COVID also plays a role in relation to the occupational situation. However, we did not explicitly consider this aspect because the study objective was oriented differently and the questionnaire was designed accordingly. The written survey used for data collection can also be regarded as a limitation. Self-reporting and the self-assessment of impressions and feelings are highly subjective and may also be perceived very differently depending on the time context. It was therefore not possible to objectively verify the information provided. The cross-sectional study design can reflect only the point in time at which the survey was conducted. Therefore, changes over time cannot be represented and no conclusions can be drawn regarding cause-and-effect relationships. Furthermore, the present study was not designed to test or validate specific theories in the field of organizational psychology. Therefore, it was not possible to propose explanatory mechanisms or suggest specific interventions. Although questions relating to the situation before the pandemic may provide indications regarding developments over time, they may nevertheless be influenced by recall bias. This can affect the accuracy of the data, which could compromise the validity of the study. Longitudinal studies would be more suitable for observing changes over time. Finally, because the study population was recruited through an accident insurance institution with an insured population consisting predominantly of women, men are underrepresented in our study and no generally applicable conclusions can be drawn regarding gender-specific findings.

## 5 Conclusion

The COVID-19 pandemic has made substantial changes also in the occupational context. Personnel in medical professions in particular showed a significant deterioration in their assessment of work ability compared with the pre-pandemic situation. Their work situation was characterized by a high work–privacy conflict, high emotional demands and work-related time pressure. This was associated with higher strain and more frequent thoughts of leaving the profession than that experienced by employees in non-medical professions. The results provide a better understanding of the health impairments experienced by employees in particularly demanding sectors. In situations such as the pandemic, alongside infection control, preventing high levels of psychological stress plays an important role in maintaining and strengthening long-term mental health and preventing employees from leaving highly demanding professions.

## Data Availability

The raw data supporting the conclusions of this article will be made available by the authors, without undue reservation.
