# Supplementary material for: Effects of the COVID-19 pandemic on working conditions and mental health among employees in health and social services
Source: Front Public Health. 2026 Aug 31;14:1899434. doi: 10.3389/fpubh.2026.1899434 (PMC13572610; doi:10.3389/fpubh.2026.1899434)
Supplement: Supplementary file 1 [file Table_1.docx]

# Supplementary Table 1: Scales of the questionnaire

| **Scale** |  | **Reference** |
| --- | --- | --- |
| Sociodemographic data | Age in years,  Sex,  Housing situation (single, adults without children, adults without children),  Size and weight,  Smoking | Created by the authors |
| Pre-existing diseases | Self-diagnosis/ medical diagnosis/ no | WAI (18) |
| Individual infection history | SARS-CoV-2 infection,  Frequency of infections,  acute and persistent symptoms | Created by the authors |
| Professional information | Occupation,  Workplace,  Working hours (Full-time, part-time, other),  Employment since… (<1 year, 1-5, 6-10, >10 years) | Created by the authors |
| Change in employment status | Has your professional situation changed since the pandemic?  How has your professional situation changed since 2020? | Created by the authors |
| Work ability | Current and bevor COVID-19 pandemic  (0 – 10, very poor –very good),  In relation to physical and psychological job demands  (1 – 5, very good – very poor) | WAI (18) |
| Emotional demands | Dealing with other people's problems (1 – 5, never/hardly ever –always),  Emotionally demanding work (1 – 5, very low –very high) | COPSOQ (19) |
| Work privacy conflict | Job demands interfere with private life,  Changing private plans because of work,  Work requires a great deal of energy, negative impact on private life,  Work takes up a great deal of time, negative impact on private life  (1-5, disagree – fully agree) | COPSOQ (19) |
| Time pressure at work | Currently and before COVID-19 pandemic:  Frequent time pressure at work,  Decisions under time pressure,  Too little time for all tasks  (1-5, almost never – almost always) | COPSOQ (19) |
| Social support | Social support from colleagues and supervisors:  Colleagues/supervisors are reliable,  Discussion of problems with colleagues/supervisors,  Support from colleagues/supervisors  (1-5, almost never – almost always) | SALSA (20) |
| Occupational change options in last 12 months | Thoughts of leaving profession,  Thoughts of changing jobs  (1 – 5, never –daily) | COPSOQ (19) |
| Symptoms of depression and  anxiety | 0 – 6, ≥3 symptoms of depression or an anxiety disorder | PHQ-4 (21) |
| Health-related quality of life (HRQoL) | 0 – 100, higher scores indicate better HRQoL | VR-12 (22) |
